# Evaluation of App-Based Serious Gaming as a Training Method in Teaching Chest Tube Insertion to Medical Students: Randomized Controlled Trial

**DOI:** 10.2196/jmir.9956

**Published:** 2018-05-21

**Authors:** Patrick Haubruck, Felix Nickel, Julian Ober, Tilman Walker, Christian Bergdolt, Mirco Friedrich, Beat Peter Müller-Stich, Franziska Forchheim, Christian Fischer, Gerhard Schmidmaier, Michael C Tanner

**Affiliations:** ^1^ Heidelberg Trauma Research Group Center for Orthopedics, Trauma Surgery and Spinal Cord Injury, Trauma and Reconstructive Surgery Heidelberg University Hospital Heidelberg Germany; ^2^ Department of General, Visceral and Transplantation Surgery Heidelberg University Hospital Heidelberg Germany

**Keywords:** games, experimental, education, professional, general surgery, emergency medicine, problem-based learning, chest tubes, simulation training, clinical competence

## Abstract

**Background:**

The insertion of a chest tube should be as quick and accurate as possible to maximize the benefit and minimize possible complications for the patient. Therefore, comprehensive training and assessment before an emergency situation are essential for proficiency in chest tube insertion. Serious games have become more prevalent in surgical training because they enable students to study and train a procedure independently, and errors made have no effect on patients. However, up-to-date evidence regarding the effect of serious games on performance in procedures in emergency medicine remains scarce.

**Objective:**

The aim of this study was to investigate the serious gaming approach in teaching medical students an emergency procedure (chest tube insertion) using the app Touch Surgery and a modified objective structural assessment of technical skills (OSATS).

**Methods:**

In a prospective, rater-blinded, randomized controlled trial, medical students were randomized into two groups: intervention group or control group. Touch Surgery has been established as an innovative and cost-free app for mobile devices. The fully automatic software enables users to train medical procedures and afterwards self-assess their training effort. The module chest tube insertion teaches each key step in the insertion of a chest tube and enables users the meticulous application of a chest tube. In contrast, the module “Thoracocentesis” discusses a basic thoracocentesis. All students attended a lecture regarding chest tube insertion (regular curriculum) and afterwards received a Touch Surgery training lesson: intervention group used the module *chest tube insertion* and the control group used *Thoracocentesis* as control training. Participants’ performance in chest tube insertion on a porcine model was rated on-site via blinded face-to-face rating and via video recordings using a modified OSATS tool. Afterwards, every participant received an individual questionnaire for self-evaluation. Here, trainees gave information about their individual training level, as well as previous experiences, gender, and hobbies. Primary end point was operative performance during chest tube insertion by direct observance.

**Results:**

A total of 183 students enrolled, 116 students participated (63.4%), and 21 were excluded because of previous experiences in chest tube insertion. Students were randomized to the intervention group (49/95, 52%) and control group (46/95, 48%). The intervention group performed significantly better than the control group (Intervention group: 38.0 [I_50_=7.0] points; control group: 30.5 [I_50_=8.0] points; *P*<.001). The intervention group showed significantly improved economy of time and motion (*P*=.004), needed significantly less help (*P*<.001), and was more confident in handling of instruments (*P*<.001) than the control group.

**Conclusions:**

The results from this study show that serious games are a valid and effective tool in education of operative performance in chest tube insertion. We believe that serious games should be implemented in the surgical curriculum, as well as residency programs, in addition to traditional learning methods.

**Trial Registration:**

German Clinical Trials Register (DRKS) DRKS00009994; https://www.drks.de/drks_web/navigate.do?navigationId=trial.HTML&TRIAL_ID=DRKS00009994 (Archived by Webcite at http://www.webcitation.org/6ytWF1CWg)

## Introduction

### Clinical Background

No matter what the underlying cause, to increase the benefit for the patient, the insertion of a chest tube should be as quick and accurate as possible [1]. Therefore, profound anatomical knowledge, meticulous positioning, and standardized execution are crucial for the success of this therapy [1]. Due to the proximity of thoracic and abdominal organs, incorrect insertion of a chest tube might lead to potentially lethal complications [2]. Therefore, comprehensive training and assessment before an emergency situation are essential in acquiring proficiency in chest tube insertion (CTI) [1]. Over the last years, the education of junior doctors and medical students has become more diverse, voluminous, and challenging. Therefore, there is an increasing need for comprehensive, objective, and resource-sparing educational concepts that ensure a high-quality education of standardized procedures without impairing patient safety [3]. In particular, early and decisive training using an educational tool might standardize execution of CTI and ultimately improve emergency care of traumatized patients and patients’ outcome [1].

### Current Training Methods

Training methods for CTI cover theoretical instructions, the use of animal models, and teaching with real patients supervised by an experienced surgeon. However, these established training methods have significant limitations. First, this type of education is limited by an increasing shortage of manpower in hospitals [4,5], and second, teaching students and inexperienced doctors with the help of real patients is not always possible with regards to patient safety. Third, not all facilities have the capability of employing animal models. Additionally, these methods show limited objectiveness in the assessment of learning success, resulting in an inaccurate educational quality [6,7]. Therefore, novel training methods are needed that enable students and inexperienced doctors to study a procedure realistically and furthermore, self-assess their performance without the need of a supervising surgeon. Additionally, future training methods should aim at providing highest levels of education without endangering patient safety.

### Serious Games for Teaching Medical Students

Serious games have become more prevalent in surgical training of physicians and medical students because of their ability to increase intrinsic motivation [8,9]. They enable students to study and train a procedure without need for a supervising surgeon. In addition, errors that are made during training with serious games have no effect on patients, and patient safety might be improved. Thus, because of the omnipresence of computer games in student’s lives, the focus in educational research has shifted towards serious games in context with medical training [9-12].

In a systematic review by Graafland et al published in 2012 [13], the authors stated that simulation and serious gaming represent ideal teaching methods for optimization of residents’ knowledge and skill before they are entrusted with procedures performed on real patients [13]. They also concluded that serious games may be used to train everyday clinical abilities such as surgical procedures [13]. In a consecutive study by Graafland et al [14], the authors examined the rate of acceptance among surgical educators and trainees regarding serious games as a training method. They found that serious games were viewed as positive by 78% of the participants, and 66% of the participants would play the game in their leisure time [14].

Defining serious games remains challenging as several classifications exist [15]. The first formal definition of the concept was provided by Abt in 1970, where he presented simulations and games to improve education. Since then, multiple classifications, both market- and purpose-based, have been postulated, and in 2002, Sawyer redefined the definition of serious games based on his idea of connecting a serious purpose to knowledge and technology from the video game industry [15]. In 2011, Djaouti et al [15] established a novel and comprehensive classification that combined the analysis of both “serious” and “game” dimensions: the gameplay/purpose/scope model [15]. Applying the classification onto the training app Touch Surgery (TS; G: type: game-based; Goals: match; Means: move, select / P: educative message broadcasting, mental training / S: market: health care; Target audience: medical students, physicians) identifies it as a serious game [15].

Validation of TS has been performed before for various specialist fields. Sugand et al (2015) published results regarding the use of TS in intramedullary femoral nailing [16]. They demonstrated construct, content, and face validity of the intramedullary nailing module. Additionally, the authors stated that TS could be used to allow orthopedic trainees to learn operative steps and be subsequently tested before and after surgery [16]. TS was also evaluated positively by a study of Paro et al [17], who used TS in the context of open carpal tunnel release. In their study, they were also able to show positive results regarding construct, face, content, and acceptability validity of TS [17]. Moreover, TS has been validated for cognitive training and assessment of laparoscopic cholecystectomy [8,9], as well as for orbital floor reconstruction [18].

### Objective

Up-to-date evidence regarding the effect of serious games on performance in procedures in trauma surgery and emergency medicine remains scarce. In this study, we sought to determine the influence of serious gaming on surgical training in CTI.

## Methods

### Study Design

The study was conducted from April 2017 to July 2017 at the Center for Orthopedics, Trauma Surgery and Spinal Cord Injury, Trauma and Reconstructive Surgery at the University of Heidelberg. We implemented the validated surgical training app TS (Kinosis Ltd, London, United Kingdom) [14] in a standardized and structured setting into medical student education to complement existing training methods and reduce necessary training staff and resources. In addition, we used an objective structural assessment of technical skills (OSATS) to advance and standardize training. The current OSATS tool for CTI was developed based on key steps of correct CTI that were modified and revised by a team of trauma and general surgeons [1]. This study was designed as a prospective, single-center, rater-blinded, two-arm, parallel group randomized controlled trial (RCT), and the study protocol was published in 2017 [1]. The trial is reported in accordance with Consolidated Standards of Reporting Trials of Electronic and Mobile HEalth Applications and onLine TeleHealth. The study was executed in concordance with the Declaration of Helsinki. Before the enrollment of participating students, approval was received from the local ethics committee of the University of Heidelberg (S-174/2016). In addition, the study was registered at the German Clinical Trials Register (DRKS) before commencement of the study (DRKS0009994). No changes to the trial design were performed after the commencement of the trial.

### Randomization

To minimize selection bias, a blocked randomization, stratified by gender, was utilized to randomly assign (1:1 ratio) participants into either an intervention group or a control group. Randomization was performed at the beginning of day 1 before the lecture and theoretical introduction. An independent employee using sealed opaque envelopes performed randomization [1]. The same employee assessed whether students complied with the study instructions before assessment—otherwise this employee was not involved in any other aspects of the study.

### Primary End Points

The primary end point of this study was the operative performance during CTI based on the standardized and evaluated OSATS scoring tool as measured by direct observation of a blinded rater during the course [1].

### Secondary End Points

Secondary end points of this study were the operative performance during CTI based on the standardized and evaluated OSATS scoring tool as measured by indirect video observation of two independent and blinded raters, as well as combined ratings of indirect and direct raters. Additionally, differences between on-site and video ratings were evaluated. Furthermore, analysis of the correlation of extracurricular activities and hobbies with operative performance was performed, as well as a time-dependent performance analysis. Another secondary end point was the subgroup analysis regarding gender-dependent differences in the operative performance [1]. There were no changes to the primary and secondary trial outcomes after commencement of the study.

### Sample Size Determination

To detect differences with a significance level Cronbach alpha=.05 and a power of 1-β=0.8, a group size of N=45 was determined in a sample size determination performed before this study [1].

### Statistical Analysis

Before the statistical analysis, all data were completely anonymized. Statistical analyses were carried out by SPSS statistics version 24.0 (IBM Corp). For analysis of nonparametric, nonrelated data, the Mann-Whitney *U* test was carried out. Correlation analysis for gender influences, as well as influence of leisure activities on operative performance, was calculated via Spearman correlation coefficient. Intraclass correlation coefficient for interrater reliability (IRR) between the “indirect” raters was measured via two-way random absolute agreement intraclass correlation analysis. For all tests, a *P* value less than .05 was considered statistically significant. Data is expressed as median values (xMed) and interquartile ranges (I_50_). The presentation of the results is done via box and whisker plots.

### Participants

According to our inclusion criteria, only medical students enrolled at the medical faculty of the University of Heidelberg during their clinical years (3rd-6th year) who reached the age of 18 years were included in the study. Participants having previous clinical experience and practice regarding CTI were excluded from this study. Participation in the study was offered as a voluntary training opportunity for CTI in context with the regular surgical curriculum. All participants had access to the app during the study because of provision of iPads with preloaded app and module. Each participating student received information about the study before participation. Furthermore, informed consent for anonymous data collection, as well as anonymous recording of videos during the training sessions, was obtained for each participant.

### Materials

#### The Utilized Serious Games

**Figure 1:**
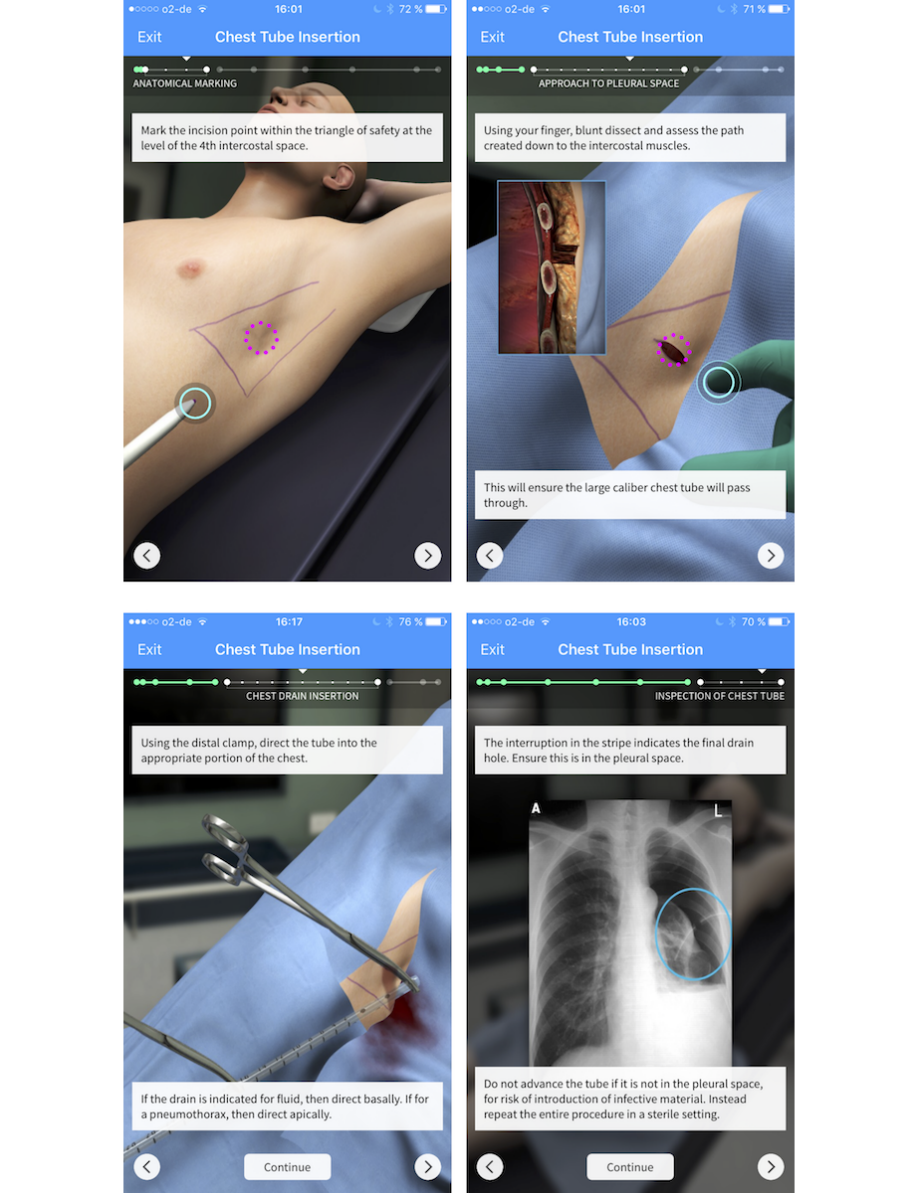
Screenshots of the Touch Surgery “Chest Tube Insertion” module. Panels A to D visualize the different key steps of the module. A: anatomical location of the safe surgical approach; B: illustration of the correct subcutaneous preparation; C: handling of instruments and tube; D: radiological control using a chest X-ray.

TS [19] has been established as an innovative and cost-free app for mobile devices [8,16] and can be downloaded from Google Play and iPhone operating system (iOS, Apple Inc) stores. The software enables users to train medical procedures in a rendered three-dimensional environment and then guides users through every stage of each procedure using touchscreen motion gestures. Hereafter, users can self-assess their training effort via active rehearsal of the steps of the procedure [20]. All participants used version 4.14.6, and there were no changes in content of the modules used during the course of the study. Currently, procedures in TS are divided into modules (over 100 procedures can be trained), and users learn procedure-specific steps such as patient positioning and access to the operating field [8]. Hereafter, in training mode, the app leads the user through each relevant step of the operation, instrument selection, and application in the specific operative procedure. Afterwards, users can switch to the self-assessment part of the software, and each step is assessed by multiple-choice questions training “cognitive decision making” [8]. The module “Chest tube insertion” was developed by Rafael J. Grossmann, an attending surgeon at Eastern Maine Medical Center, and consists of visualization of the safe zone for insertion, correct handling, and right sequence of instruments used, as well as positioning of the chest tube (Figure 1).

**Figure 2:**
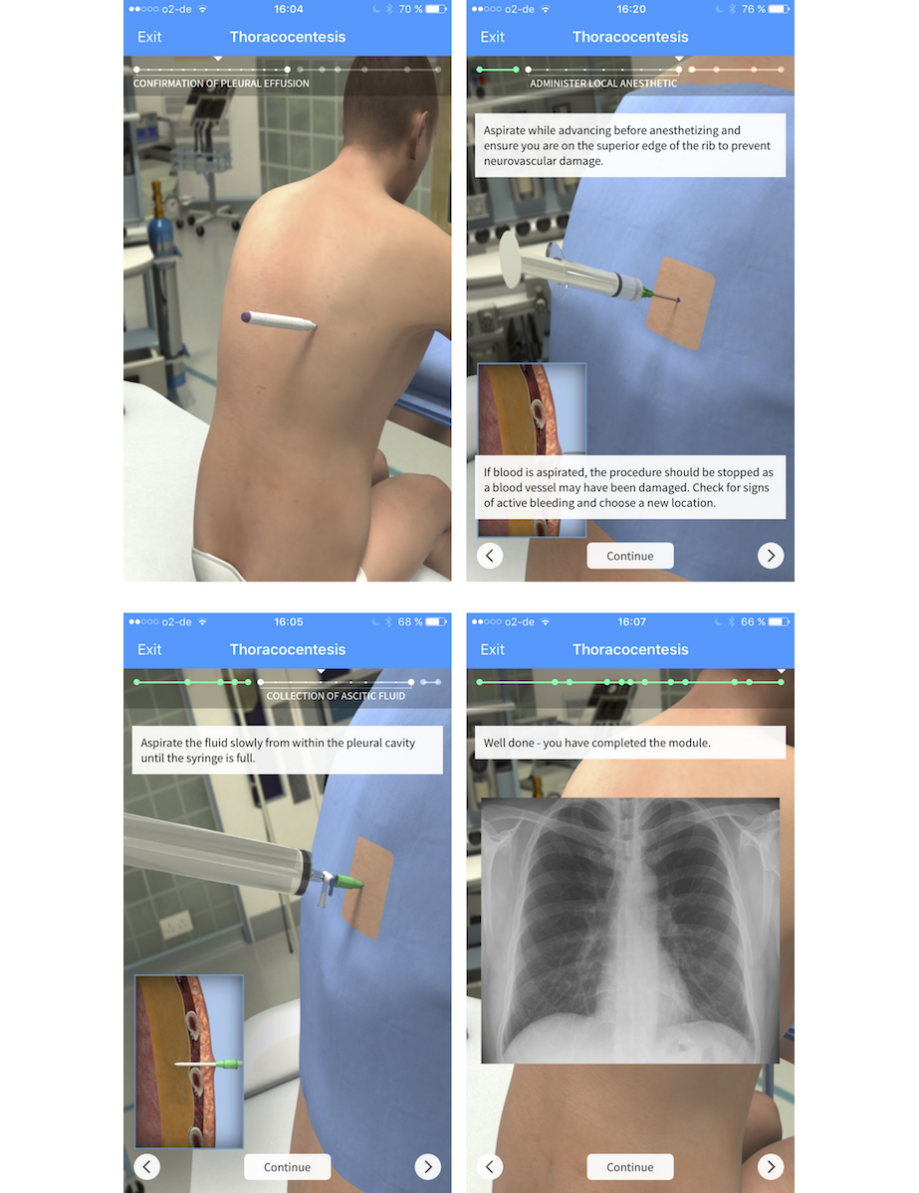
Screenshots of the Touch Surgery “Thoracocentesis” module. Panels A to D visualize the different key steps of the module. A: anatomical location of approach; B: correct administration of local anesthetics; C: aspiration of fluid; D: radiological control using a chest X-ray.

Therefore, the module teaches each key step in the insertion of a chest tube and enables users the meticulous application of a chest tube. In contrast, the module “Thoracocentesis” was developed by Shannon Toohey, a clinical instructor in the Emergency Department at UC Irvine, and discusses a basic thoracocentesis. The key steps: confirmation of pleural effusion using chest X-ray, anesthesia with local anesthetic, and pleural effusion aspiration are taught, and the handling of the used instruments is visualized (Figure 2).

This module was chosen because of its similarities in presentation, while allowing enough differentiation of the key aspects of OSATS score without risk of obfuscation. In addition, because of both a different approach and a different set of instruments, as well as a profoundly different procedure, both modules differ considerably, and thoracocentesis can be used as a control procedure without confounding the results of the study.

#### The Training

**Figure 3:**
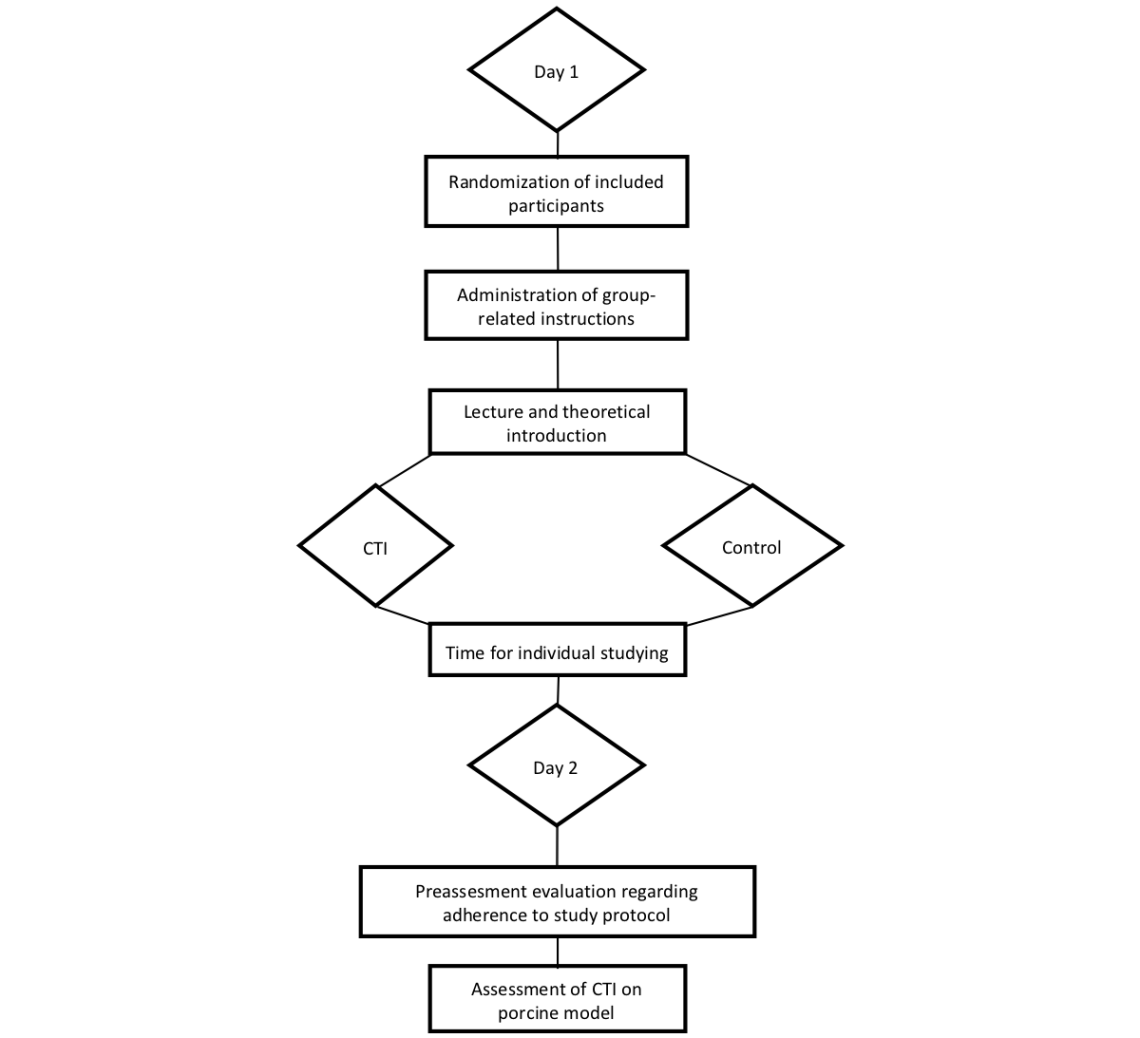
Visualization of the time schedule of the training and assessment part of this study. Randomization was performed before the first study-related interventions, and group-related instructions were given to individual participants. Introduction of both groups via a lecture occurred on the first day between 09:00 and 11:00 AM. In the afternoon (2:00-4:00 PM) of the same day, training using the app Touch Surgery was conducted under supervision of an experienced trauma surgeon. Afterwards, the rest of the day was free to provide enough time to self-study as instructed. At the beginning of day 2, the unblinded employee in charge of randomization assessed whether participants complied with the given instructions. Afterwards, operative performance was assessed utilizing the porcine model via a blinded on-site rater. CTI: chest tube insertion.

Training was conducted in context with the regular surgical curriculum. Participation in the study was voluntary. Subsequent to randomization, all participants received structured instructions regarding their respective training curriculum. In particular, participants of the control group were encouraged to further self-study using literature and available books, whereas participants of the intervention group were advised not to do so. Hereafter, all participants received the standardized theoretical training regarding CTI as part of a theoretical lecture by an experienced surgeon [1]. Participants were given access to the script of the lecture and information on where to find further material for self-studying CTI (eg, books and e-learning material). At the end of the lecture, the teaching surgeon instructed participants to further self-study the topic of the lecture as per initially delivered instructions. Thereby, participants of the control group were instructed to self-study CTI (topic of lecture), whereas participants of the intervention group received initial information not to do so regardless of instructions given at the lecture. Therefore, training in the control group was equivalent to the standard surgical education regarding CTI in our institution. Hereafter, students were introduced into the app-based serious games and the handling of TS [1]. On the afternoon of day 1, training with the app-based serious games was conducted in a training lesson lasting 120 min, supervised by experienced surgeons not involved in the randomization or analysis of study results. While participants of the intervention group used the module “Chest Drain Insertion” (Figure 1) for training of CTI, the participants of the control group used the module “Thoracocentesis” (Figure 2). Training, regardless of group, was conducted until participants reached an overall app-based score of 95% on performance of the assigned module. This was intended to assure trainees had performed the module while leaving enough margin for differentiation of scores during OSATS rating. Otherwise, participants in the control group attended every introduction and assessment included in the training sequence. A supervising experienced surgeon monitored performance of the assigned training modules to ensure adherence to each group-specific protocol [1]. Therefore, the intervention period was short, reducing the attrition bias [21]. Hereafter, participants had the rest of the day free for self-study, if applicable, as per initial instructions. Thereby, enough time was given to the participants to work through the provided material and further self-study CTI (Figure 3).

#### The Assessment

**Figure 4:**
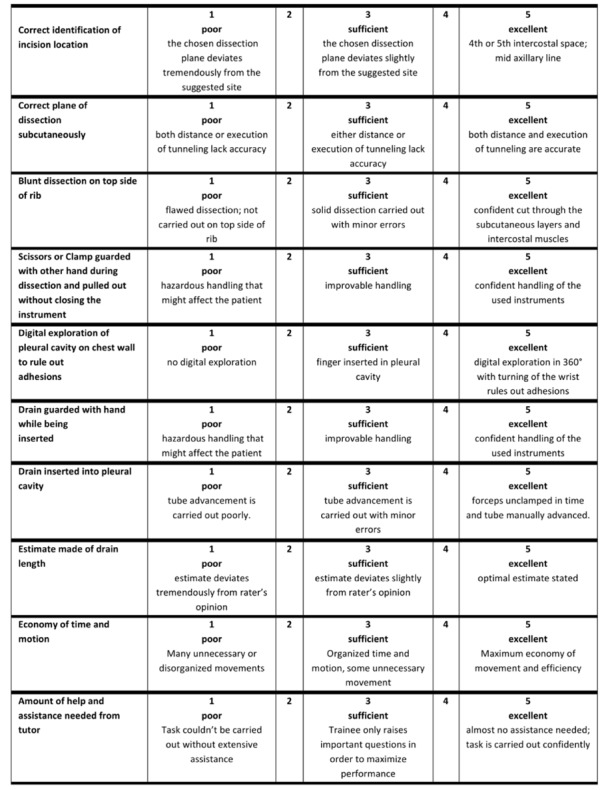
Visualization of the modified objective structural assessment of technical skills (OSATS) score for chest tube insertion (CTI).

The OSATS for CTI was developed based on the CTI Scoring System by Hutton et al [22]. In particular, the OSATS was based on key steps of correct CTI that were modified and revised by a team of trauma and general surgeons [1,22]. As shown in Figure 4, the score consists of 10 key steps. Each key step is scored from 1 (worst) to 5 (best), based on a 5-point Likert scale [1]. The maximum possible score was 50 points in total, the minimal score was 10 points (Figure 4).

Before assessment, the intervention of interest was purposely not clarified, and participants were solemnly instructed to comply with their initially delivered instructions. At the beginning of the second day, participants were asked if they complied with their respective instructions (Figure 3). In particular, participants of the control group were asked if they had adequately studied CTI, and participants of the intervention group were asked whether they had not conducted further self-study. Participants that failed to comply with the instructions would have been excluded from the study (all participants complied with the instructions in our study). During assessment session, participants performed a CTI on a previously prepared porcine model [1]. A blinded on-site rater evaluated the performance of participants face-to-face using the modified OSATS tool (Figure 4) for CTI. Performance was videorecorded, showing only the porcine model and the hands of the participants. Furthermore, the on-site rater supervised the use of instruments and use of personal safety equipment, thereby guaranteeing participants’ safety during the course of the study. Afterwards, two independent blinded video raters performed a blinded, video-based evaluation using the same scoring tool [1]. All raters were experts from the Center for Orthopedics, Trauma and Reconstructive Surgery and Spinal Cord Injury, Heidelberg University. Afterwards, every participant received a personal questionnaire for self-evaluation. Herein, trainees gave information about their individual training level, as well as previous experiences, gender, and hobbies (see Multimedia Appendices 1 and 2) [1].

## Results

### Participants

**Figure 5:**
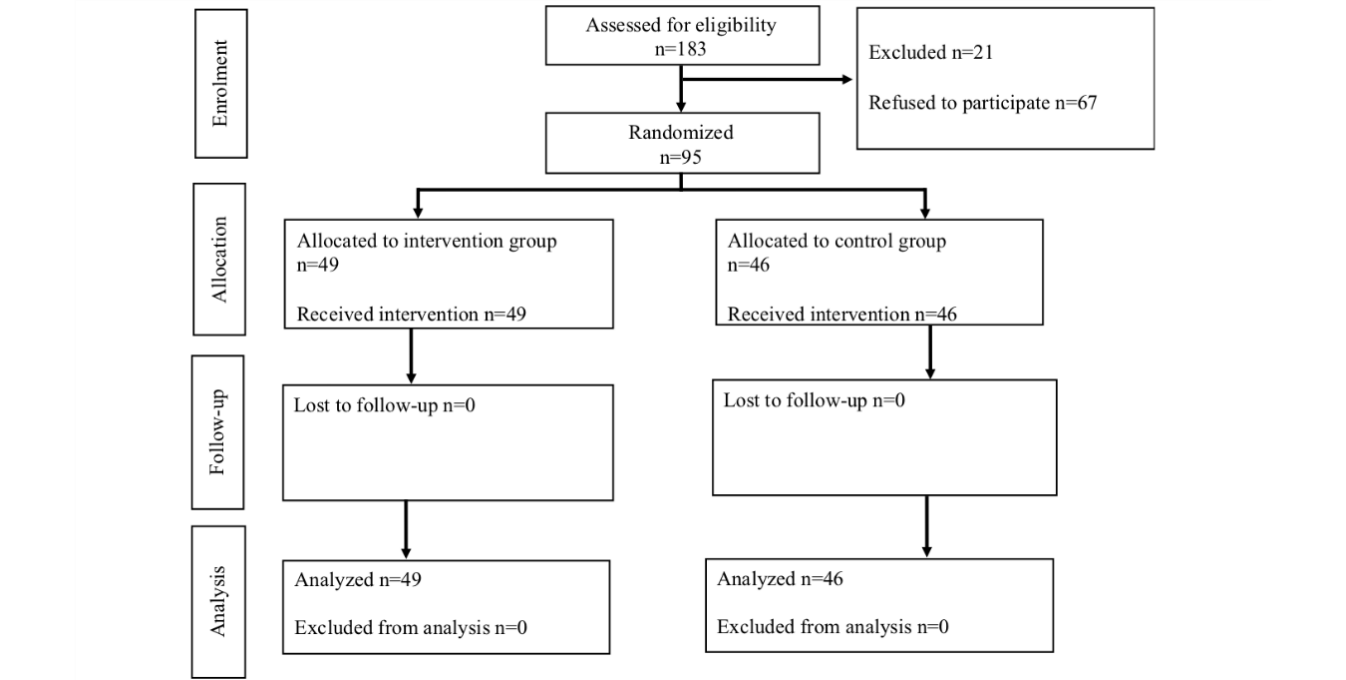
Study flowchart based on Consolidated Standards of Reporting Trials (CONSORT) guidelines.

From April 2017 to July 2017, 183 students enrolled in the emergency medicine course of our hospital, of which 116 students participated (63.4%) in this study; 21 participants had to be excluded from the analysis because of previous experiences in CTI (Figure 5).

Students were randomly assigned to the intervention group (49/95, 52%) and control group (46/95, 48%). All students participated in the clinical part of the medical curriculum (Table 1), and most students (84%, 80/95) were in their 6th semester. For the intervention group, the median age of participants was 22.0 years (I_50_=1.0), and 33 participants were female (67%, 33/49). In the control group, the median age of participants was also 22.0 years (I_50_=3.0), and 27 participants were female (59%, 27/46). Further data regarding the demographics of participants are depicted in Table 1.

**Table 1:** Participants’ demographics.

Characteristics		Intervention group (N=49)	Control group (N=46)
**Sex, n (%)**			
	Male	16 (33)	19 (41)
	Female	33 (64)	27 (59)
**Age (years), median (I_50_)**		22.0 (1.0)	22.0 (3.0)
	Male	22.0 (2.0)	22.0 (3.0)
	Female	22.0 (1.0)	22.0 (2.0)
**Leisure activity, n (%)**			
	Playing video games (total)	38 (78)	38 (83)
	Playing an instrument (total)	31 (63)	34 (74)
	Regular sportive activity (total)	43 (88)	39 (85)
	Previous experience in handicraft work (total)	24 (49)	31 (67)
**Level of education (semester), n (%)**			
	5	1 (2)	0 (0)
	6	41 (84)	39 (85)
	7	0 (0)	1 (2)
	8	7 (14)	5 (11)
	10	0 (0)	1 (2)

### Primary End Point

#### Operative Performance in Direct Observation

**Figure 6:**
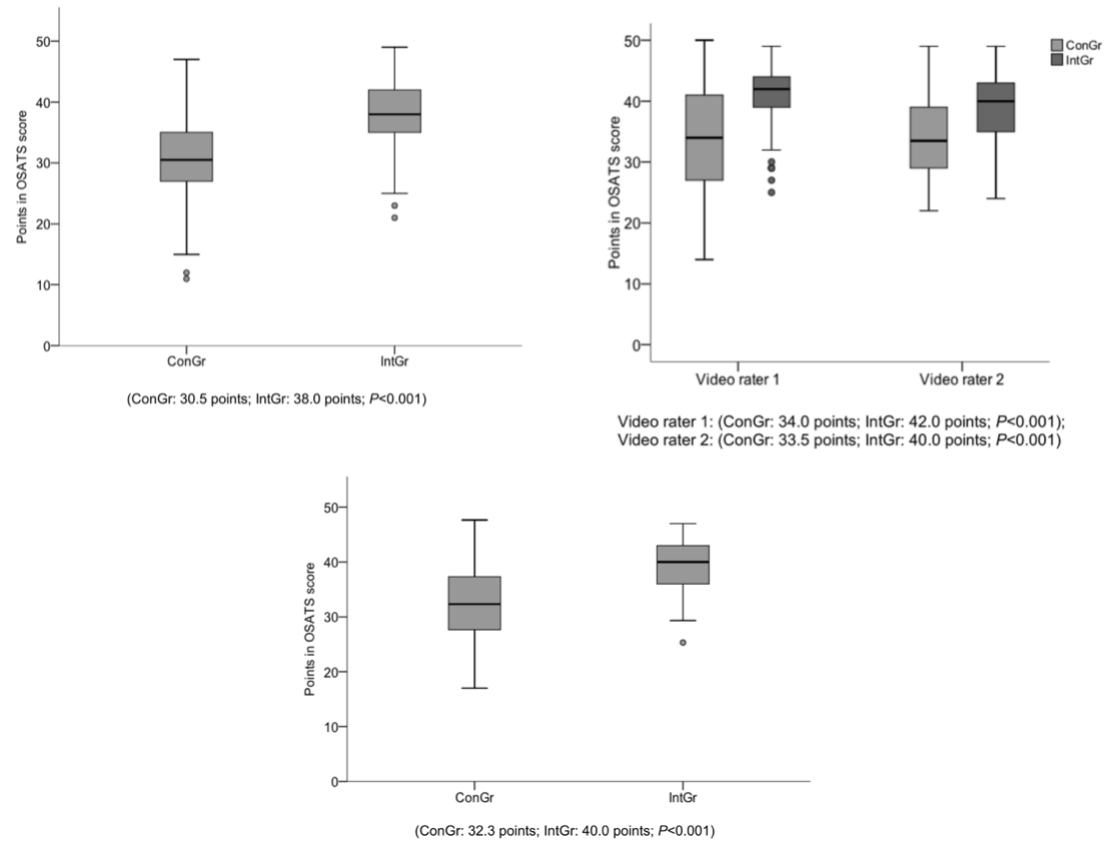
Outcome of operative performance. A: box plot showing points reached in direct objective structural assessment of technical skills (OSATS) by intervention and control group. B: box plot showing points reached in “indirect” rating by intervention and control group. C: box plot showing points reached in average of all three ratings by intervention and control group. ConGr=control group and IntGr=intervention group.

The primary end point of this study was the influence of serious games (TS) on the operative performance in CTI evaluated by the OSATS scoring tool measured via direct observation of a blinded rater during the course. Analysis of the data revealed that participants of the intervention group performed significantly better than participants of the control group in general (intervention group: 38.0 [I_50_=7.0] points; control group: 30.5 [I_50_=8.0] points; *P*<.001; Figure 6), as well as in each key step besides the “Correct plane of dissection subcutaneously.”

Participants of the intervention group showed a significantly improved economy of time and motion (intervention group: 4.0 [I_50_=1.0] vs control group: 3.0 [I_50_=1.0]; *P*=.004) and needed significantly less help from the supervising surgeon (intervention group: 4.0 [I_50_=1.0] vs control group: 2.0 [I_50_=1.0]; *P*<.001). Furthermore, participants of the intervention group were more confident in the handling of the required instruments (intervention group: 3.0 [I_50_=2.0] vs control group: 3.0 [I_50_=2.0]; *P*<.001) than participants of the control group, and the digital exploration of the pleural cavity was performed significantly better (intervention group: 4.0 [I_50_=2.0] vs control group: 2.0 [I_50_=2.0]; *P*<.001; Table 2). Median time of performing a CTI was 4:15 min in the intervention group. Participants of the control group needed a median of 4:17 min.

**Table 2:** Results of an objective structural assessment of technical skills (OSATS) subgroup analyses for direct rating.

Step of chest tube insertion	Intervention group, xMed (I_50_)	Control group, xMed (I_50_)	*P* value
Correct identification of incision location	5.0 (0.0)	5. (1.0)	.01^a^
Correct plane of dissection subcutaneously	4.0 (1.0)	3.0 (1.0)	.06
Blunt dissection on top side of rib	5.0 (1.0)	4.0 (2.0)	.02^a^
Scissors or clamp guarded with other hand during dissection and pulled out without closing the instrument	3.0 (2.0)	2.0 (1.0)	.03^a^
Digital exploration of pleural cavity on chest wall to rule out adhesions	4.0 (2.0)	2.0 (2.0)	<.001^a^
Drain guarded with hand while being inserted	4.0 (2.0)	3.0 (2.0)	<.001^a^
Drain inserted into pleural cavity	4.0 (1.0)	3.0 (1.0)	<.001^a^
Estimate made of drain length	5.0 (2.0)	3.0 (4.0)	<.001^a^
Economy of time and motion	4.0 (1.0)	3.0 (1.0)	.004^a^
Amount of help or assistance needed from tutor	4.0 (1.0)	3.0 (1.0)	<.001^a^
Total	38.0 (7.0)	30.5 (8.0)	<.001^a^

^a^A *P* value less than .05 is considered as statistically significant.

### Secondary End Points

#### Operative Performance in Indirect Observation

Operative performance based on indirect video observation revealed significant differences between groups. In particular, participants of the intervention group performed significantly better than participants of the control group regardless of the video rater (rater 1: intervention group: 42.0 [I_50_=7.0] vs control group: 34.0 [I_50_=14.0]; *P*<.001; rater 2: intervention group: 40.0 [I_50_=9.0] vs control group: 33.5 [I_50_=10.0]; *P*<.001; Figure 6). Interestingly, participants of the intervention group were significantly better in the handling of the scissors and clamps during direct observation compared with the control group (intervention group: 3.0 [I_50_=2.0] vs control group: 2.0 [I_50_=1.0]; *P*=.03), whereas indirect observation revealed better tendencies without statistical significance (intervention group: rater 1: 4.0 [I_50_=1.0], rater 2: 4.0 [I_50_=1.0] vs control group: rater 1: 3.0 [I_50_=2.0], rater 2: 3.0 [I_50_=1.0]; rater 1: *P*=.098, rater 2: *P*=.38). Overall, indirect ratings revealed smaller differences between groups than the direct rating. The analysis of the IRR revealed an excellent correlation between the results from both indirect raters (correlation index: 0.929 [95% CI 0.894-0.953]) [23]. Furthermore, analysis between direct and indirect rating revealed a good correlation as well (correlation index: 0.723 [95% CI 0.423-0.858]). In addition, combined ratings of direct and indirect observation confirmed statistically significant differences (intervention group: 40.0 [I_50_=7.2] vs control group: 32.3 [I_50_=10.1]; *P*<.001; Figure 6).

### Influence of Gender and Hobbies on Operative Performance

Gender of participants did not correlate with operative performance during CTI in the total study collective. Regular sportive activity had a significant positive correlation with the operative performance during CTI regardless of prior training (Spearman index: .214; *P*=.04). However, positive correlation of regular sportive activity was higher in the control group compared with the intervention group (Table 3). Furthermore, previous experience in handicraft work had no correlation with operative performance in the intervention group (Pearson index: −.071; *P*=.63), whereas within the control group, participants that had previous experience in handicraft work performed significantly better than participants that did not (Pearson index: .353; *P*=.02; Table 3). Somewhat surprisingly, the data from this study showed that playing video games had no correlation with operative performance during CTI in the total study collective (Spearman index: −.007; *P*>.95; Table 3).

### Evaluation of Touch Surgery as a Serious Gaming Device by Participants

Participants were asked to evaluate TS as a serious gaming device after performing CTI. Participants rated TS as an efficient training device, and when asked if they would continue to train with TS, the majority responded positively. In addition, participants were asked to evaluate both serious gaming and traditional learning methods (lecture) regarding the training benefit, level of simulation regarding the reality, and benefit regarding the handling of operative situations. Analysis of our data revealed that participants scored serious games better than traditional learning methods in all aspects regardless of the module that was trained (Table 4).

**Table 3:** Correlation regarding the influence of leisure activities on the operative performance.

Type of leisure activity	Total study collective		Intervention group		Control group	
	Spearman index	*P* value	Spearman index	*P* value	Spearman index	*P* value
Regular sportive activity	.214	.038^a^	.129	.379	.287	.05
Previous experience in handicraft work	.055	.599	−.071	.631	.353	.02^a^
Playing video games	−.007	.949	−.073	.621	−.045	.78

^a^A *P* value less than .05 is considered as statistically significant.

**Table 4:** Participants’ evaluation of training.

Factors of evaluation	Lecture, xMed (I_50_)	Training with Touch Surgery, xMed (I_50_)	*P* value
Training benefit	3.0 (2.0)	2.0 (1.0)	<.001^a^
Level of simulation regarding the reality	4.0 (2.0)	2.0 (1.0)	<.001^a^
Benefit regarding the handling of operative situations	4.0 (3.0)	3.0 (2.0)	<.001^a^

^a^A *P* value less than.05 is considered as statistically significant.

## Discussion

### Principal Findings

In this study, we sought to determine the influence of serious games on surgical training of CTI. We utilized the validated surgical training app TS in addition to a modified OSATS in an RCT. The results from this study indicated that serious gaming using a relevant content might be superior to traditional teaching methods including training with serious games with an irrelevant content regarding the operative performance in CTI. Participants from this study evaluated TS as an efficient and motivating training device. Furthermore, most participants wanted to continue the training with TS.

The results from this study support previous findings [24-26]. Participants that trained with TS showed an improved operative performance when placing a chest tube both in direct and indirect evaluation by blinded raters using the OSATS scoring tool. Participants of the intervention group performed significantly better in key steps regarding patient safety. In particular, participants of the intervention group were more accurate in the digital exploration of the pleural cavity and needed significantly less help from the supervising surgeon. Therefore, training with TS might help in enabling students to perform surgical procedures with a higher degree of independency.

Participants of the intervention group performed significantly better in the handling of the scissors and clamps. This is noteworthy because of the fact that TS offers merely gesture-based controls of the instruments used in the respective module of the app and only visualization of their correct use. Differences in the handling of instruments might be caused by differences in mental practice. Mental practice utilizes the systemic use of mental imagery to rehearse an action before the actual performance without the need for physical movement [27,28]. Mental practice has been validated as an efficient training method in teaching laparoscopic surgery [27], as well as in learning basic surgical skills [29]. In training mode, TS instructs and visualizes the correct use of instruments regarding the necessary action, whereas in test mode, the user needs to utilize gesture-based controls to initiate the use of instruments. Therefore, the user needs to visualize the necessary action using the required instrument before each respective task of the procedure. This visualization process might induce mental practice [8]. Participants of the control group trained with a different set of instruments, and therefore, visualization of the correct use of the required instruments for CTI was missing. Thus, better performance in the use of the actual instruments might be because of stimulation of mental practice by use of TS.

In addition, mental practice has been successfully implemented in teaching and rehearsing complex psychomotor tasks in several domains such as sports or music [28]. Our data indicated that sports activity positively correlates with operative performance. However, this correlation was more distinct in the control group. Participants that exercise often might show better performance in mental practice and therefore benefit more from a theoretical lecture, whereas use of TS with relevant content might induce mental practice regardless of previous experiences in sports activities. Therefore, positive effects of sports activity regarding mental practice might be reduced in the intervention group because of induction of mental practice through the use of TS with relevant content and might explain the higher correlation of sports activity and operative performance in the control group.

The influence of gender on surgical skill acquisition is not clear yet [30]. Although studies of Schueneman et al [31] and Madan et al [32] found some differences between males and females in surgical skill acquisition [33], the findings of Kolozsvari et al [34], Grantcharov et al [35], Nickel et al [36], and Kowaleski et al [37] support the results of our study that no correlation between operative performance and gender of participants could be found.

In our literature review, we found no evidence for the influence of handicraft on surgical performance. According to our data, there was no correlation between trainees’ performance and experience in handicraft work for the intervention group, but we interestingly found a significant correlation for participants of the control group. On the basis of these results, the influence of experience in handicraft work on trainees’ performance is not clear yet. Further investigation of this question to prove or disprove the hypothesis that experience in handicraft work could influence surgical performance is therefore needed.

Finally, we examined the correlation between the operative performance of the trainees and playing video games. Influence of video gaming on surgical skills has been described in various studies [38-40]. Rosser et al [41] for instance reported better performance of participants with experience in playing video games when training laparoscopic interventions. It should be noticed that in most cases, the influence of video gaming was proven for training laparoscopic interventions. In contrast to these findings, the study of Khatri et al [42] found no correlation between video gaming and surgical performance for orthopedic skill acquisition when examining dynamic hip screw simulation. Those findings support our study results. We found no correlation for both groups between playing video games and surgical performance in CTI. Regarding these results, we suppose that the influence of video gaming on the surgical performance depends on the type of surgical intervention. It might be assumable that, in contrast to laparoscopic interventions, video gaming has much lower influence on surgical performance on interventions in trauma surgery.

Participants from this study evaluated TS as an efficient and motivating training device. Furthermore, most participants wanted to continue the training with TS. According to Hutchinson et al, motivation to learn can be intrinsic (from the trainee) and extrinsic (from external influences). Intrinsic factors can be improvement of personal achievement, preparation for new situations, fun, and competition [43]. Serious gaming with TS might increase the intrinsic motivation of students by offering educational modules while providing a fun experience and because of the self-assessment, instant feedback regarding improvement. Therefore, implementation of TS in surgical education might increase students’ motivation to self-educate.

### Limitations

Despite relevant findings of this study regarding the training benefit of serious games with a relevant content in the context of CTI, our study has limitations. Participants of the control group received the initial lecture, as well as a related but irrelevant game-based study exercise. This might have led to confusion among participants of the control group leading to a poorer performance in the exercise and therefore limiting the results of the study. However, participants were instructed regarding their specific training program before the study. In addition, all students participated in the introductory lecture normally held on CTI, and students were given access to the script of the lecture and information on where to find further material for self-study (eg, books and e-learning material). Furthermore, participants of the control group were specifically interviewed before the assessment if they complied with the instructions and had adequately studied CTI. Therefore, because of the design of the study, we believe the influence of confusion derived by the control procedure and the influence of the control procedure as a distractor to be minimal. Nonetheless, the results of this study might be limited by this possibility. Before assessment, the character of the tests and the intervention of interest were purposively not clarified. However, participants of the control group were given the instruction to further study CTI, and participants of intervention group were given a single intervention. Thereby, it can be assumed that students suspected CTI to be the intervention of interest. This might limit the results of our study as participants may have performed disproportionately well by preparing especially for CTI. However, the specific type of assessment and the parameters of interest remained unknown. In addition, participants were only assessed if they complied with the instructions given at the beginning of the study. Compliance was analyzed based on subjective statements of participants before the assessment. The subjective nature of the analysis might bias the results of this study by leaving the risk of an over- or underreporting of compliance. Evaluation of motivation and satisfaction regarding serious gaming as an educational method was based on the subjective self-evaluation of the participants. It is possible that there were inaccuracies between the different groups because of incorrect answers in the participants’ self-assessment. Another limitation lies in the fact that TS is only offered in English. Some participants struggled with the language barrier and needed help in translating the different instruments. However, supervising surgeons helped students regarding difficulties in translating specific terms. Therefore, we believe that the language barrier does not influence the results of the study.

### Conclusions

The results from this study indicate that utilizing serious games with relevant content might provide a higher level of education in preparing medical students to perform CTI than traditional learning methods, including serious games with irrelevant content. Furthermore, training with serious games using a relevant content seems to improve the independency of students in performing a CTI. In addition, serious gaming might increase the intrinsic motivation of students by offering educational modules while providing a fun experience and because of the self-assessment, instant feedback regarding improvement. Thereby, implementation of TS in surgical education might increase student’s motivation to self-educate. In conclusion, the results from this study led us to believe that serious games are a valid and effective tool in the education of medical students regarding the operative performance in CTI. We believe that serious games should be implemented in surgical training curricula of medical students in addition to traditional learning methods and might add benefit to the training curriculum of residency programs. However, further studies are needed to clarify the training benefit of TS during the education of residents to confirm this assumption.

## Appendix

Multimedia Appendix 1 Questionnaires for self-assessment of students. Assessment for control group.

Multimedia Appendix 2 Questionnaires for self-assessment of students. Assessment for study group.

Multimedia Appendix 3 CONSORT‐EHEALTH checklist (V 1.6.1).

## Abbreviations

CTI: chest tube insertion
IRR: interrater reliability
TS: Touch Surgery
RCT: randomized controlled trial
OSATS: objective structural assessment of technical skills
