# Supplementary material for: Evaluation of App-Based Serious Gaming as a Training Method in Teaching Chest Tube Insertion to Medical Students: Randomized Controlled Trial
Source: J Med Internet Res. 2018 May 21;20(5):e195. doi: 10.2196/jmir.9956 (PMC5987048; doi:10.2196/jmir.9956)
Supplement: Multimedia Appendix 2 [file jmir_v20i5e195_app2.pdf]

Heidelberger Studentenkurs „Drainstudie“

1. ***Angaben zum Probanden:***
   - Alter: ....
   - Semester: ....
   - Geschlecht männlich ☐ weiblich ☐

   - Chirurgie Modul aktuell/absolviert ja ☐ nein ☐
   - Chirurgie Famulatur absolviert ja ☐ nein ☐
   - Generelle OP-Erfahrung (Selbsteinschätzung): none/ limited/ moderate/ advanced/ expert
   - Anlage Thoraxdrainage (Selbsteinschätzung): none/ limited/ moderate/ advanced/ expert
   - Geschätzte Anzahl gesehener Thoraxdrainagen Eingriffe: Total: ....

2. ***Interessen:***
   - Ich spiele oder habe regelmäßig Computerspiele gespielt (Computer, PlayStation, Nintendo, Gameboy, Handy etc.):
     Ja ☐ täglich ☐ wöchentlich ☐ seltener ☐ Nie ☐
   - Falls ja, dann überwiegend:
     ☐ Geschicklichkeit (Action, EgoShooter, Jump´n´Run, Flugsimulation, Racing)
     ☐ Kombinationsfähigkeit (Strategie, Adventure, Schach, Logische Denkaufgaben)

   - Ich spiele oder habe regelmäßig ein Instrument gespielt: Ja ☐ Nein ☐

   - Ich treibe oder habe regelmäßig Sport getrieben: Ja ☐ Nein ☐

   - Ich interessiere mich für technische Dinge und /oder bastle gerne, bzw. habe viel gebastelt:
     Ja ☐ Nein ☐

3. ***Vorerfahrung:***
   Teilnahme an einem notfallmedizinischen Trainingskurs zu einem vorherigen Zeitpunkt (z.B. Ausbildung zum Notfallsanitäter, Rettungssanitäter, Ausbildung in der Intensivmedizin etc.)

   Ja ☐ Nein ☐

   Wenn ja bitte spezifizieren...

4. ***Bewertung des Trainingsformats:***
   4.1. Bewerten Sie die folgenden Aussagen bezüglich der Nützlichkeit der **<u>Anleitung in der Vorlesung</u>** mit einer Skala von 1-5 <u>(1 trifft genau zu; 5 trifft überhaupt nicht zu</u>)

Statement	Anleitung Vorlesung
Allgemein hilfreich zum Erlernen der Anlage einer Thoraxdrainage	
Simuliert genau die Operationssituation	
Hilfreich beim Training der Instrumentenkoordination	

4.2. Bewerten Sie die folgenden Aussagen bezüglich der Nützlichkeit von **TouchSurgery für das Training der *Anlage einer Thoraxdrainage*** mit einer Skala von 1-5 (1 trifft genau zu; 5 trifft überhaupt nicht zu)

Statement	TouchSurgery
Allgemein hilfreich zum Erlernen der Anlage einer Thoraxdrainage	
Simuliert genau die Operationssituation	
Hilfreich beim Training der Instrumentenkoordination	
Hilfreich beim Training der 3D-Koordination auf einem 2D-Bildschirm	

4.3. Bewerten Sie die folgenden Aussagen mit einer Skala von 1-5 (1 trifft genau zu; 5 trifft überhaupt nicht zu)

Mein Trainingsformat hat mir Spaß gemacht	
Meine Trainingsmodalität war ein effektives Mittel zum Erlernen der Anlage einer Thoraxdrainage	
Ich würde gerne mit der gleichen Trainingsmodalität noch mehr üben	
Durch die Teilnahme an diesem Kurs fühle ich mich im Hinblick auf die zukünftige Anlage einer Thoraxdrainage gut vorbereitet	
Der Kurs hat für meine spätere Berufsausübung **keine** Relevanz	
Ich habe Interesse am Fach Chirurgie	
Durch diesen Kurs hat sich mein Interesse/ meine Motivation für das Fach Chirurgie gesteigert	
Durch diesen Kurs hat sich mein Interesse/ meine Motivation für das Fach Chirurgie **nicht** verändert	

Ich bin damit einverstanden, dass diese Daten in anonymisierter Form zu wissenschaftlichen Zwecken verwendet werden: Ja ☐ Nein ☐

Vielen Dank für die Teilnahme an der Studie und das Ausfüllen des Fragebogens! Sie leisten damit einen wesentlichen Beitrag zur Verbesserung unseres Kursangebotes.
